# Temporal relationships between BMI and obesity-related predictors of cardiometabolic and breast cancer risk in a longitudinal cohort

**DOI:** 10.1038/s41598-023-39387-w

**Published:** 2023-07-31

**Authors:** Bin Xu, Liang Lv, Xin Chen, Xingyue Li, Xunying Zhao, Huifang Yang, Wanting Feng, Xia Jiang, Jiayuan Li

**Affiliations:** 1 Department of Epidemiology and Biostatistics, Institute of Systems Epidemiology, and West China-PUMC C. C. Chen Institute of Health, West China School of Public Health and West China Fourth Hospital, Sichuan University, 16#, Section 3, Renmin Nan Lu, Chengdu, 610041 Sichuan People’s Republic of China; 2 Department of Nutrition and Food Hygiene, West China School of Public Health and West China Fourth Hospital, Sichuan University, Chengdu, China

**Keywords:** Breast cancer, Biomarkers, Cardiology

## Abstract

Prospective inter-relationships among biomarkers were unexplored, which may provide mechanistic insights into diseases. We investigated the longitudinal associations of BMI change with trajectories of biomarkers related to cardiometabolic or breast cancer risk. A longitudinal study was conducted among 444 healthy women between 2019 to 2021. Cross‑lagged path analysis was used to examine the temporal relationships among BMI, cardiometabolic risk score (CRS), and obesity‑related proteins score (OPS) of breast cancer. Linear mixed-effect models were applied to investigate associations of time-varying BMI with biomarker-based risk score trajectories. Baseline BMI was associated with subsequent change of breast cancer predictors (*P* = 0.03), and baseline CRS were positively associated with OPS change (*P* < 0.001) but not vice versa. After fully adjustment of confounders, we found a 0.058 (*95%CI* = 0.009–0.107, *P* = 0.020) units increase of CRS and a 1.021 (*95%CI* = 0.041–1.995, *P* = 0.040) units increase of OPS as BMI increased 1 kg/m^2^ per year in postmenopausal women. OPS increased 0.784 (*95%CI* = 0.053–1.512, *P* = 0.035) units as CRS increased 1 unit per year. However, among premenopausal women, BMI only significantly affected CRS (*β* = 0.057, *95%CI* = 0.007 to 0.107, *P* = 0.025). No significant change of OPS with time-varying CRS was found. Higher increase rates of BMI were associated with worse trajectories of biomarker-based risk of cardiometabolic and breast cancer. The longitudinal impact of CRS on OPS is unidirectional. Recommendations such as weight control for the reduction of cardiometabolic risk factors may benefit breast cancer prevention, especially in postmenopausal women.

## Introduction

Obesity is a major public health concern in terms of economic costs and its effect on the quality of life and health. Higher body mass index (BMI) is a risk factor for outcomes associated with lower quality of life and functional impairment, including cardiometabolic diseases (cardiovascular disease, diabetes mellitus, chronic renal failure, and other related conditions) and breast cancer, the leading cause of morbidity and mortality in women^1,2^.

Through diverse mechanisms, obesity contributes to worsened cardiometabolic health and increases rates of cardiovascular events. Several obesity-related downstream metabolic factors, including elevated blood pressure, insulin resistance, inflammation, dyslipidemia, and hyperglycemia, have been linked to atherosclerotic disease^3,4^ and largely mediate the link between obesity and coronary artery disease^5^. The changes in metabolic factors translate into cardiometabolic stress and enhanced myocardial load, including an increase in fatty acid oxidation and a decrease in glucose oxidation, and a subsequent reduction in cardiac energy, with deleterious hemodynamic consequences^6–9^. Based on this, glucolipid metabolism (insulin, glucose, triglyceride, high-density lipoprotein cholesterol), blood pressure, and central obesity (waist circumference) were combined to form the cardiometabolic risk score. Several studies show that a stronger association is obvious between increased BMI and higher breast cancer incidence, particularly in postmenopausal women^10–13^. Abnormal regulations in the blood levels of proteins caused by adipose tissue contribute to breast cancer initiation and progression through the activation of multiple signaling pathways^14^, such as overexpression of pro-inflammatory cytokines, insulin resistance, hyperactivation of insulin-like growth factors (IGFs), adipocyte-derived adipokines, hypercholesterolemia and excessive oxidative stress^15,16^. Obesity-related proteins therefore constituted an important potential predictor of breast cancer risk in addition to a history of estrogen exposure.

There are also intrinsic links between distal obesity-related health outcomes. On the one hand, several studies have provided evidence suggesting that cardiometabolic abnormalities including abdominal obesity^17^, hyperinsulinemia^18^, type 2 diabetes^19,20^, high blood pressure^21,22^, and high cholesterol^23,24^ are associated with an increased risk of breast cancer in women. Cardiometabolic abnormalities may contribute to cancer by enhancing cell proliferation and survival, as well as angiogenesis^25^. On the other hand, breast cancer survivors are at significantly higher risk of developing cardiovascular disease, partly due to the common pathway and the known associations with aging, obesity, and insulin resistance^26^. These results highlight the importance of prospective research focusing on relationships among BMI-related adverse health effects in women.

However, there is a paucity of epidemiologic data on the temporal associations of cardiometabolic markers and obesity‑related protein markers of breast cancer risk in healthy individuals. In addition, there were combinations of various obesity-related indicators reported as potential predictors of cardiometabolic risk^27–29^ and breast cancer risk^30^. Therefore, in the study reported here, we examined the temporal relations and longitudinal associations of (i) BMI and cardiometabolic risk scores (CRS) calculated by insulin, glucose, triglyceride (TG), high-density lipoprotein cholesterol (HDL-C), waist circumference (WC), and blood pressure; (ii) BMI and obesity‑related proteins scores (OPS) calculated by adiponectin (ADP), soluble leptin receptor (sOB-R), resistin (RETN), IGF-binding protein-3 (IGFBP-3) and C-reactive protein (CRP); (iii) CRS and OPS among healthy women in a breast cancer screening cohort.

## Methods

### Study design and study population

**Figure 1:**
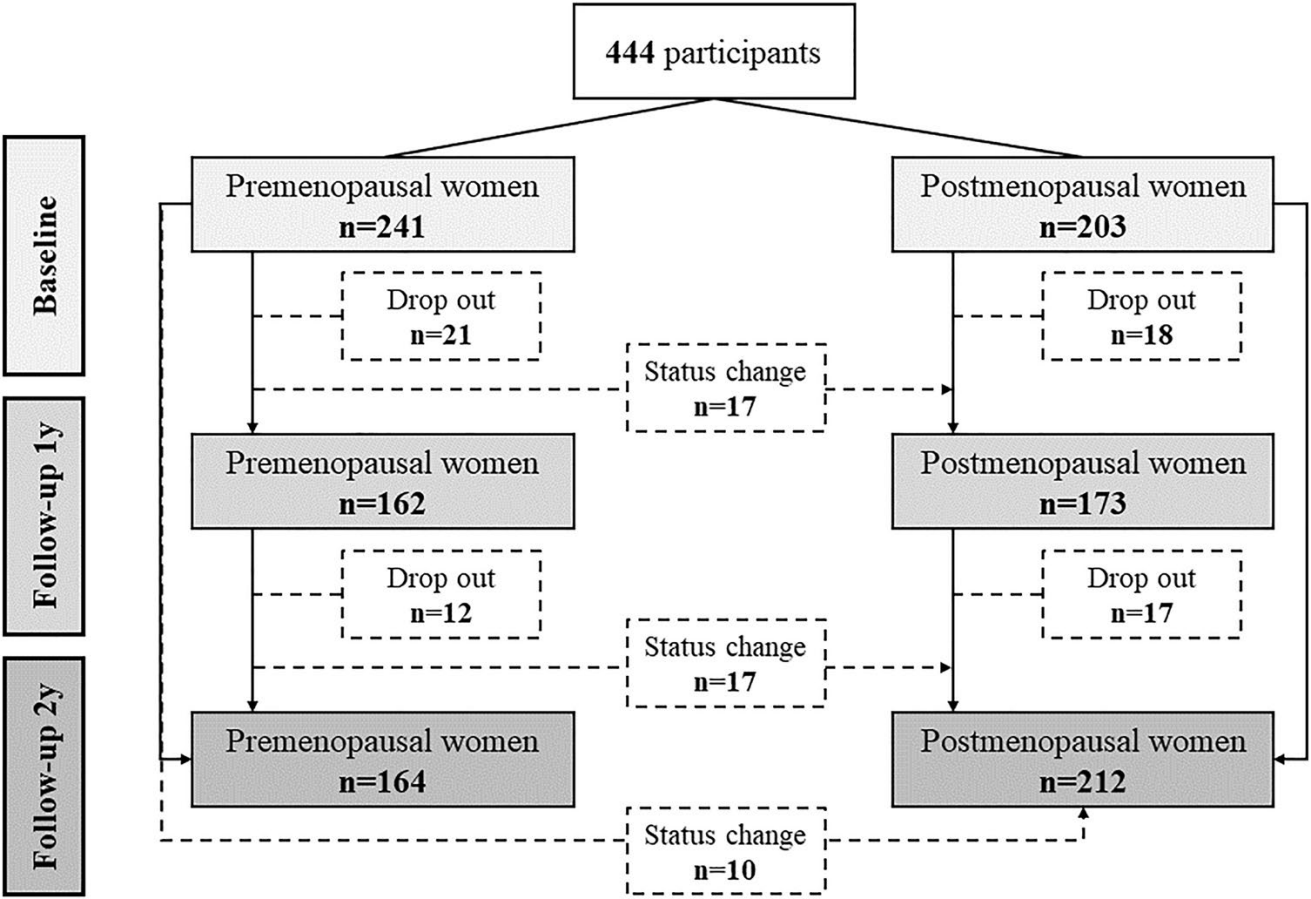
Diagram of changes in the number of participants and menopausal status from baseline to follow-up.

This longitudinal study was based on the breast screening cohort at Chengdu Shuangliu Maternal and Child Health Care Hospital, involving female participants aged 35–64 years who lived locally for at least 3 years. We excluded participants with cardiovascular diseases and malignant tumors, or who rejected to participate in the subsequent follow-up. Investigations and blood samples from eligible 444 participants were collected between May and June 2019 (as baseline). Among them, the same examinations were repeated in 335 (1-year follow-up) and 376 participants (2-year follow-up) between 2020 and 2021. Subjects who changed from pre-menopausal to post-menopausal status during follow-up (n = 44) didn’t remain in the analysis stratified by menopausal status (n = 400), but in the sensitivity analysis (n = 444), as shown in Fig. 1.

### Assessment of BMI and covariates

Anthropometric data on height and weight were measured using standard procedures by trained staff on site, and body mass index (BMI) was calculated as body weight divided by body height squared. Information on sociodemographic, menstrual, reproductive characteristics, lifestyle covariates, and medical history were collected using a structured questionnaire described previously^31^ during face-to-face interviews. Written informed consents were obtained prior to questionnaires.

### Assessment of cardiometabolic risk factors

Waist circumference was measured after expiration at the midpoint between the bottom of the rib cage and the top of the iliac crest. Blood pressure was measured on the right arm at least twice using a HEM-8613 digital monitor. The measurement protocol included, after a rest of ten minutes, three measurements in the sitting position at 2-min intervals. The mean of all three values was used as the systolic and diastolic blood pressure. Fasting venous blood samples were collected, some of which were immediately tested for blood glucose, insulin, triglycerides (TG), and HDL-C at the Laboratory of Clinical Chemistry of the site hospital, while others were centrifuged and stored at – 80 °C. A continuous cardiometabolic risk score (CRS) variable was calculated as the sum of Z-scores of waist circumference, insulin, glucose, triglycerides, HDL cholesterol, and the mean of systolic and diastolic blood pressure that are specific for the study population^27,32^. The Z-score of HDL cholesterol was multiplied by − 1, because HDL cholesterol is inversely associated with cardiometabolic. Written informed consents were obtained prior to blood samples donation.

### Assessment of breast cancer risk biomarkers

Five obesity-related biomarkers including ADP, RETN, CRP, IGFBP-3, and sOB-R were measured at the Public Health and Preventive Medicine Provincial Experiment Teaching Center at Sichuan University, and the selection procedure has been described in detail elsewhere^30,33^. The concentrations in plasma were determined by standard enzymatic methods (ELISA reagents from Wuhan Elabscience Biotechnology Co., Ltd in China), and absorbance was measured by the enzyme labeling instrument (Thermo Company, USA). Origin 9.0 software was used to obtain the standard curve and calculate specific concentrations. The pre- and post-menopausal obesity‑related protein scores (OPS) were calculated with linear‑weighted summation and the detailed formulas have been published elsewhere^30^.

### Statistical analyses

**Figure 2:**
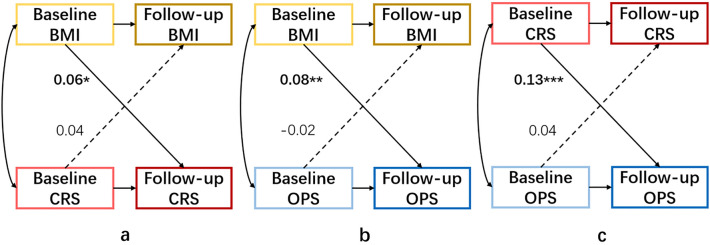
The cross‑lagged panel analysis of BMI *vs* CRS, BMI *vs* OPS, and CRS *vs* OPS. The values on the arrows are standardized path coefficients, and asterisks represent levels of p-values (*P < 0.1, **P < 0.05, ***P < 0.001). Solid arrows indicate statistically significant paths, and dashed arrows indicate statistically not significant paths.

Characteristics at baseline were compared by analysis of variance (ANOVA) (or Kruskal–Wallis test) and Chi-square tests (or Fisher’s exact test) across baseline and follow-up subgroups. To normalize skewed distributions, a natural logarithmic (ln) transformation was performed for blood glucose, insulin, TG, HDL-C, and these obesity-related proteins. We used a cross-lagged panel design to investigate the bidirectional relationship between BMI *vs* CRS, BMI *vs* OPS, and CRS *vs* OPS. Cross-lagged path analysis that examines reciprocal, longitudinal relationships among a set of inter-correlated variables was used^34^. Taking BMI *vs* CRS as an example, this method tested the effect of baseline BMI on subsequent CRS and the effect of baseline CRS on subsequent BMI simultaneously, adjusted for autoregressive effects. The cross-lag path coefficients shown in Fig. 2 were estimated simultaneously based on the correlation matrix. All parameters in the cross-lagged path analysis were estimated by constructing a structural equation model by AMOS (version 22.0). The validity of model fitting was evaluated by the goodness-of-fit index (GFI) and comparative fit index (CFI).

Further, to describe the relationships between two risk scores and the continuous BMI over time, we used the linear mixed-effect models for repeated measures (baseline and the two follow-up time points) with random intercepts. According to the menopausal status, a stratified analysis of the associations between time-varying BMI and risk scores was conducted to explore the associations in premenopausal and postmenopausal women. The details are as follows.

An interaction term between continuous BMI and time was included to capture the associations of time-varying BMI and CRS. Besides the interaction term, baseline BMI and time, the models were further adjusted for age, menopausal status, education, occupation, passive smoking, and health status at baseline to control for variation at the beginning of the study. The other BMI *vs* OPS and CRS *vs* OPS models were fitted in the same way and the sequence of independent and response variables was exchanged to assess the inverse association (CRS → OPS, OPS → CRS). We used three models to minimize the role of confounding. Model 1 was adjusted only for age (continuous variable). Model 2 was additionally adjusted for education, occupation, drinking, and smoking status (categorical variable). Model 3 was further adjusted for menarche age and number of live births (categorical variable). The results of Model 3 were considered as our primary findings, complemented by the results of Model 1 and Model 2. Finally, the relationship between the combined risk score and individual biomarkers was further explored based on Model 3 and the effect values were presented in a forest plot. All analyses were conducted using R version 4.2.0 (R Core Team (2022), Vienna, Austria.). A 2-sided P < 0.05 is considered statistically significant.

### Ethics declaration

All methods were performed in accordance with the relevant guidelines and regulations and were in accordance with the Declaration of Helsinki. The study protocol was approved by the ethics committee of West China School of Public Health and West China Fourth Hospital, Sichuan University. All subjects participated in the study voluntarily and signed informed consent forms. Confirms informed consent was obtained to publish the information/image(s) in an online open access publication.

## Results

### Characteristics of study participants

The mean (SD) age of 444 participants was 49.47 (6.36) years at baseline (Table 1). Among 241 (54%) premenopausal and 203 (46%) postmenopausal women, some had experienced a status change and dropped out, and then the distribution of menopausal status was 162 (48%) *vs* 172 (52%) at the first follow-up and 164 (44%) *vs* 212 (56%) at the last follow-up (Fig. 1). At baseline, BMI ranged from 16.66 to 35.23 kg/m^2^ with a mean (SD) of 24.31(2.89) kg/m^2^, and WC ranged from 65 to 109 cm with a mean (SD) of 83.62 (8.26) cm. Both BMI (*P* = 0.201) and WC (*P* = 0.189) were not statistically different from baseline to follow-up (Table 1). HDL-C, ADP, CRP, and s-OBR were slightly increased from baseline to follow-up (all *P* < *0.05*), while blood pressure (SBP and DBP) and other blood-based biomarkers (glucose, insulin, RETN, TG, IGFBP-3) fluctuated across the three-time points (all *P* < *0.05*).

**Table 1:** Characteristics at baseline (2019), the first follow-up (2020), and the last follow-up (2021) in the screening cohort.

Characteristic	Baseline (n = 444)	Follow-up 1y (n = 335)	Follow-up 2y (n = 376)	*P*^†^
Age, years	49.47 (6.36)	50.47 (6.47)	51.19 (6.22)	** < 0.001**
BMI, kg/m^2^	24.31 (2.89)	24.01 (2.84)	24.38 (2.85)	0.201
WC, cm	83.62 (8.26)	83.88 (8.63)	82.78 (8.69)	0.189
SBP, mmHg	133.12 (21.37)	125.29 (17.85)	132.11 (20.07)	** < 0.001**
DBP, mmHg	82.46 (12.92)	79.40 (12.06)	83.08 (13.13)	** < 0.001**
Education levels, n (%)				0.906
Primary or under	19 (4%)	13 (4%)	12 (3%)	
Middle	385 (87%)	290 (87%)	325 (86%)	
High or above	40 (9%)	32 (10%)	39 (10%)	
Occupation, n (%)				0.994
Unemployed	251 (59%)	192 (60%)	212 (59%)	
Mental worker	16 (4%)	13 (4%)	15 (4%)	
Manual worker	159 (37%)	115 (36%)	132 (37%)	
Missing	18	15	17	
Smoking, n (%)				0.368
No	437 (99%)	325 (98%)	368 (99%)	
Yes	4 (1%)	7 (2%)	5 (1%)	
Missing	3	3	3	
Drinking, n (%)				0.949
No	425 (96%)	321 (97%)	359 (96%)	
Yes	16 (4%)	11 (3%)	14 (4%)	
Missing	3	3	3	
Menopause status, n (%)				**0.009**
Premenopausal	241 (54%)	162 (48%)	164 (44%)	
Postmenopausal	203 (46%)	173 (52%)	212 (56%)	
Menarche age, n (%)				> 0.999
< 14 years old	191 (44%)	145 (44%)	164 (45%)	
≥ 14 years old	242 (56%)	184 (56%)	203 (55%)	
Missing	11	6	9	
Live birth history, n (%)				> 0.999
No	3 (1%)	2 (1%)	2 (1%)	
Yes	439 (99%)	332 (99%)	373 (99%)	
Missing	2	1	1	
Insulin, uIU/ml	1.84 [0.47]	1.78 [0.44]	1.80 [0.51]	0.181
Glucose, mmol/L	1.60 [0.19]	1.57 [0.15]	1.60 [0.15]	**0.045**
HDL-C, mmol/L	0.45 [0.19]	0.51 [0.17]	0.60 [0.19]	** < 0.001**
TG, mmol/L	0.30 [0.52]	0.41 [0.53]	0.38 [0.54]	**0.012**
RETN, ng/mL	2.17 [1.21]	2.19 [1.15]	1.69 [1.05]	** < 0.001**
CRP, ug/ml	-0.29 [1.23]	0.02 [1.21]	0.23 [1.09]	** < 0.001**
ADP, ug/ml	2.08 [0.53]	2.11 [0.54]	2.19 [0.57]	**0.010**
IGFBP-3, ng/mL	5.83 [0.81]	5.24 [0.89]	5.68 [0.80]	** < 0.001**
sOB-R, ng/mL	2.37 [0.81]	2.42 [0.70]	2.51 [0.81]	**0.037**

Values are means (SDs) for continuous variables except for plasma biomarkers, geometric means [geometrics SDs] for all plasma biomarkers, and percentages for categorical variables.

BMI, body mass index; WC, waist circumference; SBP, systolic blood pressure; DBP diastolic blood pressure; HDL-C, high density lipoprotein cholesterol; TG, triglyceride; RETN, resistin; CRP, C-reactive protein; ADP, adiponectin; IGFBP-3, insulin-like growth factor binding protein-3; sOB-R, soluble leptin receptor.

Significant values are in bold.

^†^One-way ANOVA was used for continuous variables, and Fisher's exact test was used for categorical variables.

### The temporal relationship between BMI and risk scores

556 1-year follow-up pairs from participants with complete data on BMI and biomarkers (268 women from 2019 to 2020, and 288 women from 2020 to 2021) were included in the cross-lagged path analysis. We found a positive association for the path coefficients from baseline BMI to follow-up CRS (*β* = 0.06, *P* = 0.06), but not in the reverse direction from baseline CRS to follow-up BMI (*β* = 0.04, *P* = 0.11) (Supplementary Table 1, Fig. 2a). The associations between BMI and OPS showed statistical significance for the path from baseline BMI to follow-up OPS (*β* = 0.08, *P* = 0.03), but not in the reverse direction (*β* = − 0.02, *P* = 0.28) (Supplementary Table 1, Fig. 2b). For the temporal relationship between CRS and OPS, with the increase of baseline CRS, follow-up OPS were increased (*β* = 0.13, *P* < 0.001), but CRS did not change with OPS (*β* = 0.04, *P* = 0.19) (Supplementary Table 1, Fig. 2c). All three models showed good fit (Model a: GFI = 0.970, CFI = 0.977; Model b: GFI = 0.997, CFI = 0.997; Model c: GFI = 0.997, CFI = 0.997).

### Association between time-varying BMI and risk scores

To determine how the two risk scores would be influenced by BMI change and by each other change, we further examined the associations between a time-varying exposure and outcome trajectories (exposure → outcome) (Table 2). We found 0.058 (*95%CI* = 0.009–0.107, *P* = 0.020) units increase of CRS and 1.021 (*95%CI* = 0.041–1.995,* P* = 0.040) units increase of OPS as BMI increased 1 kg/m^2^ per year in premenopausal women. OPS increased 0.784 (*95%CI* = 0.053–1.512, *P* = 0.035) units as CRS increased 1 unit per year. Among premenopausal women, after adjusting for demographic characteristics, lifestyle, and reproductive factors, only the significance of BMI → CRS remained (*β* = 0.057, *95%CI* = 0.007 to 0.107, *P* = 0.025). However, associations (BMI → CRS, BMI → OPS, CRS → OPS) were observed significantly in the fully adjusted model in postmenopausal women. No significant change of OPS with time-varying CRS (OPS → CRS) was found in both main and adjusted models.

**Table 2:** Associations between longitudinal changes in time-varying BMI and trajectories of CRS and OPS.

Outcome	Premenopausal (n = 197)			Postmenopausal (n = 203)		
	Intercept	Estimate (β, 95%CI)	P value	Intercept	Estimate (β, 95%CI)	P value
Model 1						
BMI → CRS	− 11.864	**0.058 (0.009, 0.107)**	**0.020**	− 17.440	0.045 (− 0.004, 0.095)	0.069
BMI → OPS	23.968	**1.021 (0.041, 1.995)**	**0.040**	12.012	**0.586 (0.249, 0.922)**	** < 0.001**
CRS → OPS	− 13.154	**0.784 (0.053, 1.512)**	**0.035**	8.411	**0.280 (0.029, 0.531)**	**0.029**
OPS → CRS	− 4.474	0.002 (− 0.000, 0.004)	0.115	− 4.179	0.005 (− 0.000, 0.011)	0.097
Model 2						
BMI → CRS	− 9.065	**0.056 (0.006, 0.105)**	**0.028**	− 18.798	0.049 (− 0.000, 0.099)	0.052
BMI → OPS	27.774	0.918 (− 0.090, 1.921)	0.072	22.169	**0.586 (0.243, 0.930)**	**0.001**
CRS → OPS	− 21.143	0.683 (− 0.065, 1.429)	0.073	15.826	**0.306 (0.051, 0.561)**	**0.019**
OPS → CRS	− 0.987	0.002 (− 0.001, 0.004)	0.225	− 7.502	0.006 (− 0.000, 0.012)	0.070
Model 3						
BMI → CRS	− 7.614	**0.057 (0.007, 0.107)**	**0.025**	− 20.194	**0.054 (0.004, 0.105)**	**0.033**
BMI → OPS	7.578	0.915 (− 0.090, 1.916)	0.073	14.293	**0.588 (0.238, 0.939)**	**0.001**
CRS → OPS	− 47.354	0.690 (− 0.054, 1.433)	0.069	7.744	**0.281 (0.022, 0.540)**	**0.034**
OPS → CRS	0.044	0.002 (− 0.001, 0.004)	0.225	− 9.425	0.005 (− 0.000, 0.011)	0.098

Model 1 adjusted for age at baseline.

Model 2 adjusted for age at baseline, education, career, smoking, and drinking.

Model 3 adjusted for age at baseline, education, career, smoking, drinking, menarche age, and live birth.

Significant values are in bold.

**Figure 3:**
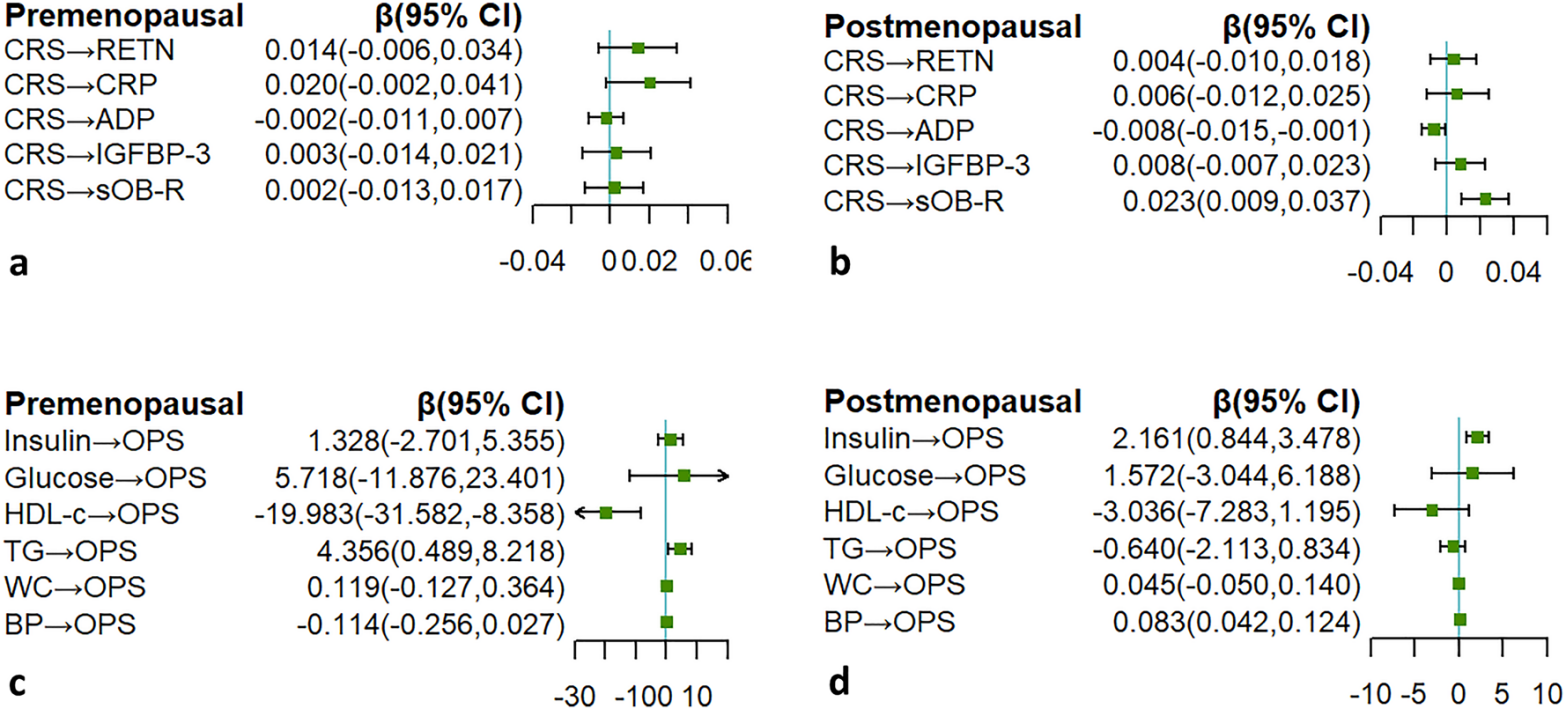
Longitudinal associations between individual biomarkers and risk scores. (**a**) Longitudinal associations between CRS and biomarkers of OPS in premenopausal women. (**b**) Longitudinal associations between CRS and biomarkers of OPS in postmenopausal women. (**c**) Longitudinal associations between OPS and biomarkers of CRS in premenopausal women. (**d**) Longitudinal associations between OPS and biomarkers of CRS in postmenopausal women.

Since the temporal relationship (CRS → OPS) was initially established, we further assessed the longitudinal effects on individual biomarkers (Fig. 3). With an increase of 1 unit per year in CRS, ADP decreased by 0.008 (*95% CI* 0.001 to 0.015) ln-units and sOB-R increased by 0.023 (*95% CI* 0.009 to 0.037) ln-units in postmenopausal women. With a ln-unit per year decrease of HDL-C or increase of TG, OPS increased by 19.983 (*95% CI* 8.358 to 31.582) and 4.356 (*95% CI* 0.489 to 8.218) units, respectively, in premenopausal women. In postmenopausal women, an increase of 1 ln-unit insulin and 1 unit blood pressure per year was associated with an increase in OPS of 2.16 (*95% CI* 0.844 to 3.478) and 0.083 (*95% CI* 0.042 to 0.124) units, respectively.

*Sensitivity analysis* based on the age-adjusted model showed consistent effects among participants, with no exclusion of those who had changed menopausal status during follow-up (Supplementary Table 2).

## Discussion

In the present longitudinal cohort study with repeatedly measured data, we demonstrated that CRS and OPS were prospectively associated with the baseline BMI, and future OPS were found to be associated with baseline CRS. Among them, associations of BMI and risk scores were replicated in the LM analysis, especially in postmenopausal women. In addition, we identified significant individual biomarkers in the CRS → OPS relationship (HDL-C, TG, ADP, and sOB-R).

The associations between adiposity and the risk of cardiometabolic disease and breast cancer were explored over the past few years, and most of these studies focused on the impact of BMI on the development of health outcome events. This study suggested that short-term changes in BMI trajectories have a significant association with risk scores of cardiometabolic and breast cancer, compensating for the inadequate capture of biomarker fluctuations at long follow-up. We used a validated continuous CRS because it is a more sensitive way to describe cardiometabolic risk than dichotomous definitions for metabolic syndrome^35,36^. Unlike metabolic diseases, there are no specific biomarkers or anthropometrics for directly characterizing breast cancer risk. We therefore use a protein score, composed of biomarkers involved in mechanisms of breast carcinogenesis including subclinical chronic low-grade inflammation and oxidative stress, anti-proliferative processes, sex hormones bio-synthesis pathway, and abnormal system and signaling of insulin resistance^30^. The score was previously established through evidence-based and experimental steps and has achieved robust predictive performance in breast cancer risk. On the other hand, there is an interplay between cardiometabolic disease and breast cancer risk, as shown in patients^26,37,38^. However, few studies prospectively explored the temporal relationship of these two risks in a cohort of healthy people based on respective biomarkers. Our study, to the best of our knowledge, presented the first repeatedly measured cardiometabolic factors and obesity-related proteins in Chinese women, which could help reveal the dynamic change of risk of cardiometabolic and breast cancer over time-varying BMI.

We found that baseline CRS was associated with follow-up OPS, and further confirmed in another analysis that the trajectory of OPS was associated with time-varying CRS. Interestingly, however, no significant association was found after exchanging the dependence of the risk scores. The temporal relationship between these two robust risk scores indicated that women at high cardiometabolic risk should pay more attention to the favorable change of obesity-related factors with the purpose of breast cancer prevention. In addition, cardiometabolic factors, as common clinical indicators, has better accessibility to identify high-risk women of breast cancer risk.

It has been reported that adiponectin possesses anti-diabetic, anti-inflammatory, and anti-atherogenic properties^39–41^. As expected, a negative correlation between adiponectin and CRS was observed in our study. Although high sOB-R downregulates the bioavailability of leptin with pro-inflammatory features^42,43^, we observed increased levels of sOB-R associated with higher CRS in postmenopausal women, seemly inconsistent with the hypothesis that this adipokine may have a favorable role in improving metabolism^44,45^. However, this negative association between sOB-R and certain cardiometabolic risk factors is so far only supported by evidence from cross-sectional studies of predominantly non-menopausal women, and more prospective studies stratified by menopausal status are needed to elucidate the mechanisms.

A meta-analysis of 26 prospective studies involving 1,628,871 women showed that TG was significantly related to breast cancer development, and HDL-C was inversely related to breast cancer risk^46^. In the present study, the associations between these two biomarkers and OPS trajectory in the same direction as previous studies were observed only in premenopausal women, highlighting the importance of implementing strategies to improve lipid levels (increase HDL-C and reduce TG) before menopause. Previous studies have found a positive correlation between insulin levels and breast cancer risk in postmenopausal women, suggesting that hyperinsulinemia is an independent risk factor for breast cancer^47^. A meta-analysis based on 18 case–control studies found an increased risk of breast cancer in women with hypertension, but only for postmenopausal women^48^. The result was consistent with the positive correlation between blood pressure and OPS found in postmenopausal women in this study.

The strength of this study is the repeated measurements of adiposity-associated biomarkers and numerous validated covariates that enabled us to evaluate the short-term prospective relationships between cardiometabolic risk and adipokine-based breast cancer risk. Leveraging the cohort study design and two types of analyses in parallel, we were able to identify robust temporal relationships. However, several limitations should be noted. First, although we have adjusted several important confounding factors, the possibility of residual confounding and measurement errors cannot be excluded. Second, our results can only explain as an association instead of a causal relationship due to the observational nature. Third, generalizability may be limited because participants in our study were predominantly screening women without cardiovascular diseases or breast cancer. However, this characteristic can also be an advantage because high compliance with screening ensures the collection of high-quality information on risk factors. When evaluating the association of adiposity with cardiometabolic risk, more studies are needed to discriminate between fat mass and lean mass components of BMI using dual X-ray energy absorptiometry^49^. The results in the present study should be further validated in other large cohorts with repeated measurement or experimental studies in the future.

## Supplementary Information

Supplementary Tables.

## Data Availability

The raw data are not publicly available due to the participant’s privacy, but derived data supporting the findings are available from the corresponding author by reasonable request.
