# Supplementary material for: Temporal relationships between BMI and obesity-related predictors of cardiometabolic and breast cancer risk in a longitudinal cohort
Source: Sci Rep. 2023 Jul 31;13:12361. doi: 10.1038/s41598-023-39387-w (PMC10390576; doi:10.1038/s41598-023-39387-w)
Supplement: Supplementary file 1 — Supplementary Tables. [file 41598_2023_39387_MOESM1_ESM.docx]

**Supplementary Table 1** Autoregressive cross-lagged temporal analyses of BMI, cardiometabolic risk score, and obesity‑related protein score

| Temporal analyses | B | β | S.E. | P |
| --- | --- | --- | --- | --- |
| **BMI & Cardiometabolic risk score** |  |  |  |  |
| **Autoregressive** |  |  |  |  |
| Baseline BMI → Follow-up BMI | 0.869 | 0.856 | 0.024 | <0.001 |
| Baseline CRS → Follow-up CRS | 0.753 | 0.733 | 0.033 | <0.001 |
| **Cross-lagged** |  |  |  |  |
| Baseline CRS → Follow-up BMI | 0.035 | 0.038 | 0.022 | 0.11 |
| Baseline BMI → Follow-up CRS | 0.068 | 0.060 | 0.036 | 0.062 |
| **BMI & Obesity‑related proteins score** | | | | |
| **Autoregressive** |  |  |  |  |
| Baseline BMI → Follow-up BMI | 0.890 | 0.876 | 0.021 | <0.001 |
| Baseline OPS → Follow-up OPS | 0.405 | 0.414 | 0.038 | <0.001 |
| **Cross-lagged** |  |  |  |  |
| Baseline OPS → Follow-up BMI | -0.002 | -0.022 | 0.002 | 0.281 |
| Baseline BMI → Follow-up OPS | 0.814 | 0.083 | 0.378 | 0.031 |
| **Cardiometabolic risk & Obesity‑related proteins score** | | | | |
| **Autoregressive** |  |  |  |  |
| Baseline CRS → Follow-up CRS | 0.781 | 0.761 | 0.028 | <0.001 |
| Baseline OPS → Follow-up OPS | 0.392 | 0.400 | 0.038 | <0.001 |
| **Cross-lagged** |  |  |  |  |
| Baseline OPS → Follow-up CRS | 0.004 | 0.036 | 0.003 | 0.192 |
| Baseline CRS → Follow-up OPS | 1.157 | 0.130 | 0.342 | <0.001 |

B indicates unstandardized regression，and β indicates standardized regression

**Supplementary Table 2** Associations between longitudinal changes in time-varying BMI and trajectories of CRS and OPS in premenopausal participants

| Outcome |  | Intercept | Estimate (β, 95%CI) | P value |
| --- | --- | --- | --- | --- |
| BMI → CRS |  | -10.545 | **0.062 (0.020, 0.105)** | **0.004** |
| BMI → OPS |  | 44.469 | **1.30 (0.448, 2.143)** | **0.003** |
| CRS → OPS |  | -11.880 | 0.626(-0.026, 1.276) | 0.060 |
| OPS → CRS |  | -3.846 | 0.002 (-0.001, 0.004) | 0.135 |
